# Molecular characterization of HIV-1 near-full-length proviral quasispecies in monocytes from patients across different virological responses

**DOI:** 10.3389/fmicb.2025.1647986

**Published:** 2025-08-21

**Authors:** Jian Li, Chuyu Zhang, Zhenyi Zou, Yawen Liang, Peng Ma, Yun Lan, Quanmin Li, Qian Kong, Ruiying He, Linghua Li, Weilie Chen

**Affiliations:** ^1^Institute of Infectious Diseases, Guangzhou Eighth People’s Hospital, Guangzhou Medical University, Guangzhou, Guangdong, China; ^2^Guangzhou Key Laboratory of Clinical Pathogen Research for Infectious Diseases, Guangzhou, Guangdong, China; ^3^Department of Clinical Laboratory, Success Hospital Affiliated to Xiamen University, Xiamen, Fujian, China; ^4^Infectious Disease Center, Guangzhou Eighth People’s Hospital, Guangzhou Medical University, Guangzhou, Guangdong, China

**Keywords:** HIV-1, quasispecies, monocytes, low-level viremia, antiretroviral therapy

## Abstract

**Introduction:**

Low-level viremia (LLV) in HIV infection, defined as detectable but low plasma viral load, is associated with an increased risk of virological failure (VF); however, the mechanisms underlying LLV remain unclear. Monocytes, as potential viral reservoirs, can migrate into tissues and differentiate into tissue-resident macrophage reservoirs, playing a critical role in viral dissemination and potentially driving persistent viremia.

**Methods:**

This study aimed to analyze and compare the molecular characteristics of near-full-length HIV-1 proviral DNA quasispecies from monocytes in three distinct virological response groups: VF, LLV, and virological suppression (VS). Genetic diversity, drug resistance mutations (DRMs), and viral tropism were assessed.

**Results:**

Of the 198 single quasispecies sequences obtained from 54 patients, 177 were identified as near-full-length genomes (NFLGs; length >8.6 kb, without inversion). The VF group demonstrated a higher prevalence of intact proviruses (82.6%) compared to the LLV (50.0%) and VS groups (22.2%). Compared to the VF group, the LLV group exhibited significantly higher hypermutation rates (42.35% vs 8.78%, *p* < 0.01) and greater median genetic distance (0.0446 vs 0.0186, *p* < 0.01). Moreover, monocytes harbored proviral DNA with DRMs that were divergent from those detected in plasma RNA. No significant differences in viral tropism were observed across groups.

**Discussion:**

Near-full-length proviral quasispecies amplified from monocytes demonstrated distinct characteristics across virological response groups. Notably, proviral quasispecies in the LLV group exhibited higher genetic diversity, suggesting unique evolutionary dynamics under low-level viral replication. These findings underscore the importance of investigating proviral quasispecies within monocytes to better understand their role in persistent HIV viremia.

## 1 Introduction

HIV-1 infection remains a significant global public health challenge. Although antiretroviral therapy (ART) effectively suppresses viral loads to undetectable levels, latent reservoirs—primarily composed of intact proviral DNA integrated into the host genome—present a major obstacle to achieving a cure. These reservoirs can reactivate upon treatment interruption, resulting in viral rebound (Finzi et al., 1999; Chun et al., 2010; Ho et al., 2013).

Even with long-term ART, some individuals exhibit persistently low levels of viral load (VL), known as low-level viremia (LLV). Persistent LLV has been identified as an independent risk factor for virological failure (VF) in previous studies (Joya et al., 2019; Li et al., 2021). According to the US Department of Health and Human Services guidelines (Department of Health and Human Services, 2024), VF is defined as the inability to achieve or maintain viral suppression, with HIV RNA levels exceeding 200 copies/mL. LLV is defined as a detectable viral load below 200 copies/mL, observed on at least two occasions, while virological suppression (VS) refers to a viral load below the lower detection limit of the assay. LLV not only elevates the risk of VF but also induces immune activation and inflammation. This immune dysregulation contributes to immune failure, elevated pro-inflammatory cytokines, and an increased incidence of non-AIDS-defining events and other adverse clinical outcomes (Crespo-Bermejo et al., 2021).

Monocytes, a key target for HIV-1, are thought to act as persistent reservoirs for the virus, although their precise role remains debated (Cattin et al., 2019; Wong et al., 2019). These cells are implicated in plasma viremia and the dissemination of HIV into anatomical sanctuaries. Monocytes are categorized into three subsets: classical (CD14^++^CD16^−^), intermediate (CD14^++^/CD16^+^), and non-classical (CD14^+^/CD16^++^), each playing distinct roles in HIV-1 infection. While classical monocytes, the most abundant subset, demonstrate resistance to HIV, the more mature CD16^+^ monocytes exhibit heightened susceptibility due to their elevated CCR5 expression (Ellery et al., 2007). Infected monocytes can migrate to tissues and differentiate into macrophages, contributing to latent reservoirs. Notably, infected monocytes can cross the blood–brain barrier (BBB), potentially facilitating viral transmission to the brain (Williams et al., 2014). Recent *in vitro* studies have confirmed that monocyte-derived macrophages harbor latent HIV reservoirs (Veenhuis et al., 2023). Furthermore, HIV DNA has been detected in monocytes, with evidence of replication capacity (Zhu et al., 2002; RifeMagalis et al., 2020). Monocytes can also carry multiple genetically distinct HIV-1 variants compared to CD4^+^ T cells. Interestingly, these variants are often identical to, or closely related to, the viral strains detected in plasma after prolonged ART (Xu et al., 2008).

During HIV-1 replication, mutations and recombination events result in the formation of a genetically diverse yet related group of viral variants within the same host, referred to as quasispecies (Wain-Hobson, 1993). This diversity enables HIV-1 to rapidly adapt to host immune pressures and antiretroviral therapies, complicating treatment strategies (Andino and Domingo, 2015). The characteristics of proviral quasispecies vary across virological response groups in HIV-1-infected individuals (Zhang et al., 2021). However, the traits of proviral quasispecies within monocytes in individuals with low-level viremia remain poorly understood.

This study analyzed and compared the genetic diversity, drug resistance mutations, and viral tropism of near-full-length proviral quasispecies in monocytes from HIV-1 infected individuals with varying virological responses. The findings reveal the molecular characteristics of proviral quasispecies in monocytes, providing new insight into the factors underlying LLV. Future research should explore how these traits can be leveraged to enhance treatment outcomes and develop novel antiviral strategies.

## 2 Materials and methods

### 2.1 Study population and sample collection

**Figure 1:**
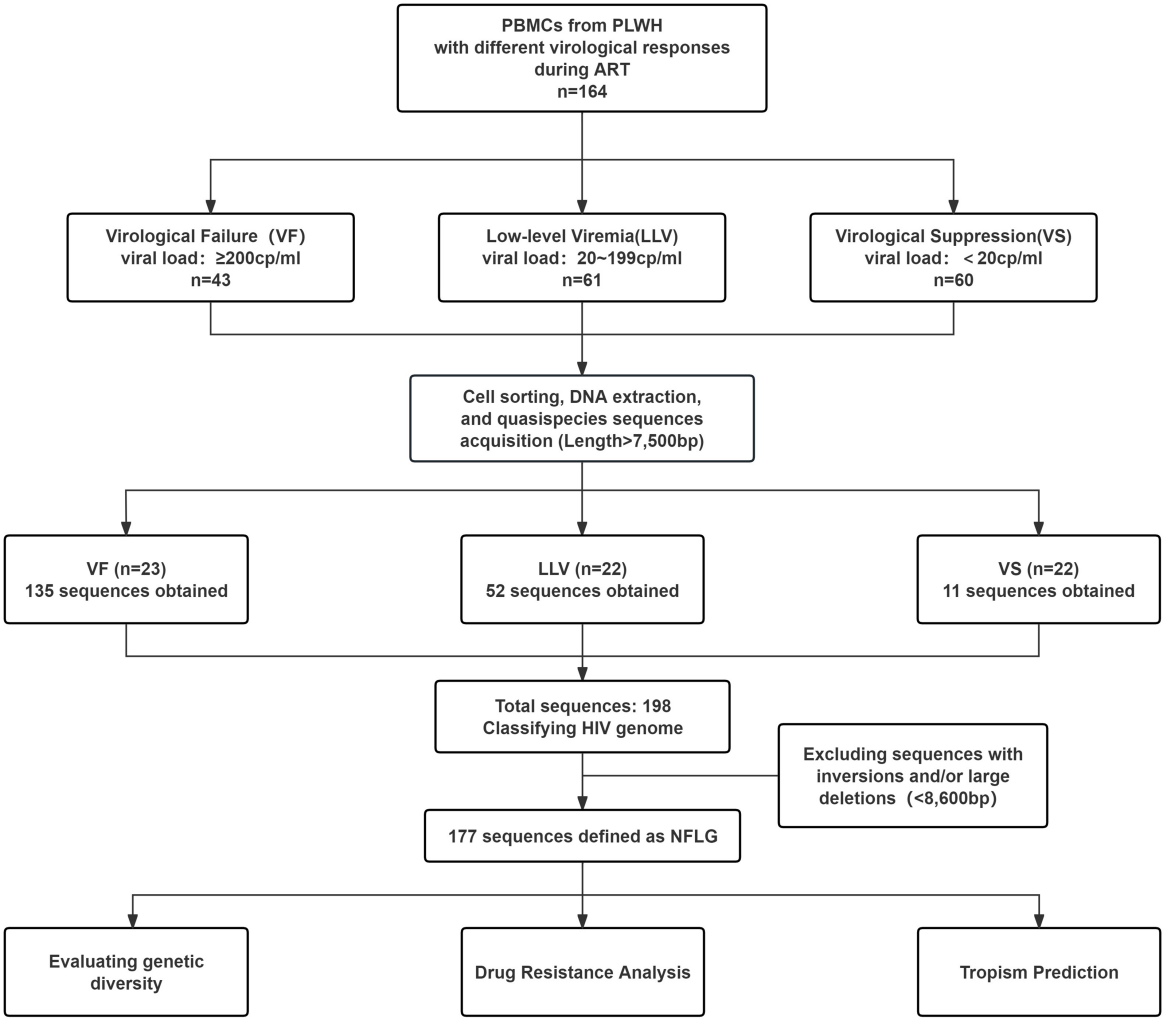
Diagram of the study design. ART, antiretroviral therapy; PLWH, people living with HIV; NFLG, near full-length genome.

Between July 2023 and July 2024, 164 people living with HIV (PLWH) attending the outpatient clinic at Guangzhou Eighth People’s Hospital were recruited. Inclusion criteria were: age ≥18 years, ART duration ≥24 weeks, and at least two viral load records. PLWH were categorized into VF (VL ≥ 200 copies/mL; *n* = 43), LLV (20 ≤ VL < 200 copies/mL; *n* = 61), and VS (VL < 20 copies/mL; *n* = 60) groups (Figure 1). Epidemiological data (date of birth, gender, high-risk behaviors) and clinical data (HIV diagnosis date, baseline viral load, CD4^+^ T cell count, CD8^+^ T cell count, and treatment regimen) were collected. Written informed consent was obtained, and 10 mL of peripheral blood was collected from each participant.

### 2.2 Isolation of monocytes and DNA extraction

Peripheral blood mononuclear cells (PBMCs) were isolated from whole blood using density gradient centrifugation. CD14^+^ monocytes were positively selected from PBMCs using magnetic beads (Miltenyi Biotec). Genomic DNA was extracted from monocytes using the TIANamp Genomic DNA Kit (Tiangen Biotech) following the manufacturer’s protocol.

### 2.3 Amplification and sequencing of near-full-length-genome

To amplify near-full-length HIV-1 quasispecies sequences, quantitative PCR (qPCR) targeting the gag region was performed using the limiting dilution method. The optimal template concentration was determined as the dilution at which 30% of the PCR reactions were positive (Hiener et al., 2017). This method, optimized from previous studies (Bruner et al., 2016), involved diluting samples to the endpoint concentration for the first round of nested PCR. Primers used included forward primer OuterF (5′-AAATCTCTAGCAGTGGCGCCCGAACAG-3′) and reverse primer OuterR (5′-TGAGGGATCTCTAGTTACCAGAGTC-3′). Each polymerase chain reaction consisted of 2 μL of diluted DNA, 1 μL of PrimeSTAR GXL DNA Polymerase (Takara), 0.3 μM of forward and reverse primers, 4 μL of 2.5 mM dNTPs (Takara), 10 μL of 5 × PrimeSTAR GXL Buffer (Takara), and 30 μL of sterile, nuclease-free water, making a total volume of 50 μL. The thermal cycling conditions were as follows: an initial denaturation at 94°C for 1 min 10 s, followed by 30 cycles of denaturation at 94°C for 20 s, annealing at 62°C for 15 s, and extension at 68°C for 10 min, with a final extension at 68°C for 10 min. The first-round nested PCR products served as templates for quadruplex qPCR (Q4PCR) to simultaneously detect four probes targeting distinct HIV regions: packaging signal (PS), gag, pol, and env (Gaebler et al., 2019). The products exhibiting positive for at least two of the four probes were inferred to represent near-full-length proviral DNA and subsequently used as templates for the second-round nested PCR. The primers used were InnerF (5′-ACAGGGACCTGAAAGCGAAAG-3′) and MSR5 (5′-GCACTCAAGGCAAGCTTTATTGAGGCT-3′). The reaction components and cycling conditions for the second round were identical to the first. PCR products were analyzed using 0.7% agarose gel electrophoresis, and correctly sized products (7,500–10,000 bp) were selected for Sanger sequencing. Sequencing data were processed (edited, trimmed, and assembled) using Sequencher 5.4.6.

### 2.4 HIV-1 genome classification

**Figure 2:**
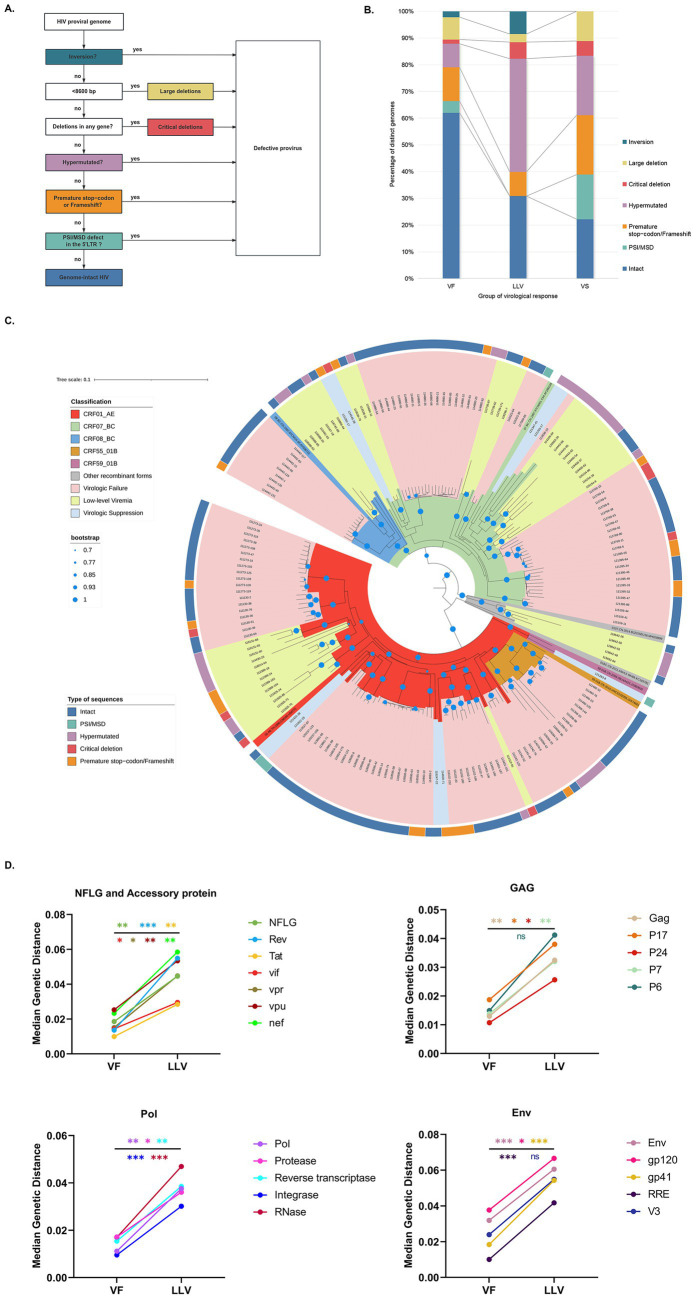
Landscape and genetic characterizations of proviral DNA. **(A)** Schematic representation of identifying defective and intact proviruses. **(B)** Proportion of distinct genomes. **(C)** Phylogenetic tree of NFLGs. Phylogenetic tree including all distinct NFLG (excluding quasispecies with inversions and large deletions). The outer circles indicate each quasispecies-associated HIV-1 genome classification. **(D)** Comparison of median genetic distances for each gene between VF and LLV. Asterisks indicate statistical significance: ns, not significant (*p* > 0.05); *p < 0.05; **p < 0.01; ***p < 0.001; ****p < 0.0001. Moreover, the color of each asterisk matches the corresponding gene in the legend.

Proviral genomes were classified into categories as shown in Figure 2A (Lambrechts et al., 2023): (i) Inversion: Detected during sequence alignment, these regions do not match the HXB2 reference sequence. (ii) Large Deletion: Defined as an internal deletion reducing the sequence length to <8,600 bp. (iii) Critical Deletion: Characterized by a start or stop codon in any gene containing a deletion. (iv) Hypermutated: Identified using the Los Alamos National Laboratory HIV Sequence Database’s ‘Quality Control’ webtool.^1^ (v) Premature Stop Codon/Frameshift: Includes stop codons or frameshift mutations caused by insertions/deletions in essential genes (gag, pol, or env). Insertions/deletions >49 nt in gag or pol and >99 nt in env are also included. (vi) PSI/MSD Defects: Includes deletions >7 bp in the PSI region (SL1 [HXB2: 691–734], SL2 [HXB2: 736–754], SL3 [HXB2: 766–779], and SL4 [HXB2: 790–810]) stem loops or mutations in the MSD site (GT, HXB2: 744–745) and cryptic donor site (GT, HXB2: 748–749). (vii) Intact: Proviruses with no defects from the above categories, potentially replication-competent.

### 2.5 Phylogenetic analysis

Genetic subtypes of quasispecies were determined using the ‘HIV BLAST’ webtool.^2^ Reference sequences obtained from the HIV sequence database^3^ were aligned with near-full-length genomes (NFLGs; excluding those with “Inversion” or “Large Deletion”) using the MUSCLE algorithm in MEGA 11.0.13. A neighbor-joining phylogenetic tree was constructed using the Kimura 2-parameter model with 1,000 bootstrap replicates and visualized using Interactive Tree of Life (iTOL, version 7). Gene-specific sequences were extracted using the ‘Gene Cutter’ webtool.^4^ Genetic distances for individual gene sequences were calculated using the Kimura 2-parameter model with 1,000 bootstraps in MEGA 11.0.13.

### 2.6 Analysis of resistance mutations

The obtained sequences were submitted to the Stanford HIV Drug Resistance Database^5^ to identify drug resistance mutations (DRMs) and evaluate antiretroviral drug resistance susceptibility. Resistance levels were classified into five categories: susceptible, potential low-level resistance, low-level resistance, intermediate-level resistance, and high-level resistance. In this study, any resistance classified as low-level or higher was considered drug resistance.

### 2.7 Viral tropism prediction

The “Gene Cutter” online tool was employed to extract nucleotide and amino acid sequences of the V3 loop. Coreceptor usage in the V3 region was analyzed using three computational genotypic tools: Geno2Pheno (with a false positive rate cutoff of 10%),^6^ WebPSSM[x4r5],^7^ and HIVcoPRED.^8^ Final coreceptor usage predictions were determined by synthesizing results from these methods. If an individual harbored viruses with different tropisms, they were classified as X4/R5 dual-tropism cases.

### 2.8 Statistical analysis

Statistical significance was determined using two-tailed tests with a threshold of *p* < 0.05. Analyses were conducted using SPSS software (IBM SPSS Statistics, version 20.0). Age comparisons utilized one-way analysis of variance and unpaired t-tests. The Kruskal-Wallis and Mann–Whitney tests were applied to compare parameters such as ART initiation delay, ART duration, baseline CD4^+^ T cell count, baseline CD8^+^ T cell count, baseline CD4/CD8 ratio, and genetic distance. Fisher’s exact test, Chi-square test, or G-test were used to analyze gender, genetic subtype, transmission route, sequence category, viral tropism, and amino acid distribution in the V3 loop. For multiple comparisons, *p*-values were adjusted using the Benjamini-Hochberg method to control the false discovery rate (FDR).

## 3 Results

### 3.1 Patient characteristics

In this study, flow cytometry was used to assess the purity of monocytes isolated by magnetic bead sorting, as well as the proportion of CD4^+^ T lymphocytes within the sorted population. The purity of CD14^+^ cells could reach over 95%, while CD4^+^ T lymphocytes consistently constituted less than 1% of the population. Among the 164 samples collected, 54 yielded near-full-length proviral genomes. The amplification success rates were as follows: VF group 53.5% (23/43), LLV group 36.1% (22/61), and VS group 15% (9/60; Figure 1). Among these 54 PLWH (Table 1), most were male (51/54), with a mean age of 46.8 years (±13.8 SD). The median time from antibody confirmation to treatment initiation was 28.5 days (IQR 12–124.5), and the median ART treatment duration was 2,225 days (IQR 1036–2,892). The LLV group was significantly older (52.3 ± 14.0 years) than the VF group (41.4 ± 13.0 years; *p* = 0.010). The VF group exhibited significantly lower CD4^+^ T cell counts (*p* < 0.05) and CD4/CD8 ratio (*p* < 0.001) than other groups. No significant differences were observed in genetic subtype (*p* = 0.100), ART duration (*p* = 0.182), or baseline CD4/CD8 ratio (*p* = 0.086).

**Table 1:** Demographic and clinical characteristics of the study participants.

Characteristic	Total (*n* = 54)	Virological response			*p*-value
		VF (*n* = 23)	LLV (*n* = 22)	VS (*n* = 9)	
Age (years), mean ± SD	46.8 **±** 13.8	41.4 **±** 13.0	52.3 **±** 14.0	47.3 **±** 10.7	0.027
Gender, n (%)					0.378
Male	51 (94.4)	21 (91.3)	22 (100)	8 (88.9)	
Female	3 (5.6)	2 (8.7)	0	1 (11.1)	
Time from HIV confirmation to sampling(days), median (IQR)	2,367 (1,059, 3,564)	2,216 (1,062, 3,375)	2,367 (720.3, 3,629)	3,647 (1725, 5,193)	0.292
Delay of ART initiation (days), median (IQR)	28.5 (12, 124.5)	25 (13, 100)	24 (11.5, 212)	59 (8.5, 1,375)	0.748
Time on ART (days), median (IQR)	2,225 (1,036, 2,892)	2,127 (778, 2,817)	2,296 (717, 2,691)	2,958 (1,647, 4,019)	0.182
HIV Subtype, n (%)					0.100
CRF01_AE	22 (40.7)	12 (52.2)	6 (27.3)	4 (44.4)	
CRF07_BC	21 (38.9)	7 (30.4)	10 (45.4)	4 (44.4)	
CRF08_BC	5 (9.3)	1 (4.4)	4 (18.2)	0	
CRF55_01B	3 (5.6)	3 (13.0)	0	0	
CRF59_01B	1 (1.8)	0	0	1 (11.2)	
Others	2 (3.7)	0	2 (9.1)	0	
Transmission category, n (%)					0.978
Homosexual transmission	18 (33.3)	7 (30.4)	8 (36.4)	3 (33.3)	
Heterosexual transmission	29 (53.7)	12 (52.2)	11 (50.0)	6 (66.7)	
Injection drug use	5 (9.3)	3 (13.0)	2 (9.1)	0	
Others/unknown	2 (3.7)	1 (4.4)	1 (4.5)	0	
Initial ART regimen, n (%)					0.898
NRTI+NNRTI	35 (64.8)	16 (69.6)	12 (54.6)	7 (77.8)	
NRTI+INSTI	13 (24.1)	4 (17.4)	7 (31.8)	2 (22.2)	
NRTI+PI/r	4 (7.4)	2 (8.7)	2 (9.1)	0	
Others	2 (3.7)	1 (4.3)	1 (4.5)	0	
Current ART regimen, n (%)					0.121
NRTI+NNRTI	20 (37.0)	9 (39.1)	5 (22.7)	6 (66.7)	
NRTI+INSTI	19 (35.2)	6 (26.1)	10 (45.5)	3 (33.3)	
NRTI+PI/r	14 (25.9)	8 (34.8)	6 (27.3)	0	
Others	1 (1.9)	0	1 (4.5)	0	
At baseline					
CD4 + T cell total count (cells/μL), median (IQR)	142.5 (57.25, 254.5)	111 (18.75, 289.8)	161.5 (90.25, 211.3)	212.5 (70, 382.5)	0.693
CD8 + T cell total count (cells/μL), median (IQR)	729 (452.5, 1,138)	851.5 (239, 1,179)	799 (649.3, 1,230)	597.5 (209.5, 1,021)	0.359
CD4+/CD8 + T cell count, median (IQR)	0.173 (0.083, 0.330)	0.127 (0.047, 0.339)	0.160 (0.082, 0.246)	0.349 (0.186, 0.657)	0.086
At the time of sampling					
CD4 + T cell total count (cells/μL), median (IQR)	417 (214.5, 608)	222 (115.5, 536)	468 (345.5, 628.3)	596 (365, 877.5)	0.010
CD8 + T cell total count (cells/μL), median (IQR)	804 (594.5, 1,052)	914.5 (582, 1,401)	805.5 (532.8, 1,041)	662 (628, 810.5)	0.252
CD4+/CD8 + T cell count, median (IQR)	0.483 (0.299, 0.809)	0.277 (0.187, 0.474)	0.535 (0.393, 1.082)	0.723 (0.641, 1.301)	<0.0001

### 3.2 Proviral quasispecies sequence landscape

In total, 198 single proviral quasispecies sequences were obtained, of which 177 NFLGs remained after excluding those with internal inversions and large deletions. The VF, LLV, and VS groups contributed 124, 44, and 9 NFLGs, respectively (Supplementary Table S1). The median number of NFLGs per PLWH was 4 (IQR 2–9) in the VF group, 1 (IQR 1–3) in the LLV group, and 1 (IQR 1–1) in the VS group. The prevalence of PLWH with intact proviral sequences in monocytes was significantly higher in the VF group (82.6%, 19/23) compared to the LLV group (50%, 11/22) and VS group (22.2%, 2/9; *p* = 0.004). No PSI/MSD defects were observed in the LLV group, and inversions were absent in the VS group. The proportion of hypermutated sequences induced by APOBEC-3G/3F was significantly higher in the LLV group compared to the VF group (42.35% vs. 8.78%, *p* = 0.008). Furthermore, the VF group exhibited a significantly higher proportion of intact proviral sequences compared to the other two groups (*p* < 0.05; Figure 2B).

### 3.3 Evolutionary distance of different virologic response

HIV BLAST analysis indicated consistent genetic subtypes among quasispecies within individuals, with CRF01_AE (40.7%) and CRF07_BC (38.9%) being the most common, followed by CRF08_BC (9.3%) and CRF55_01B (5.6%). In the neighbor-joining phylogenetic tree of NFLGs, sequences from each individual clustered by subtype without abnormal distributions, although the LLV group exhibited longer branch lengths (Figure 2C). Since only a single NFLG was obtained from some PLWH, precluding the calculation of intra-individual genetic distances of quasispecies sequences, median genetic distances were compared only between the VF (*n* = 19) and LLV (*n* = 10) groups. The LLV group had significantly higher median genetic distances (0.0446, IQR 0.0351–0.0522) than the VF group (0.0186, IQR 0.0122–0.0285, *p* = 0.004). Genetic distances were higher across all genes in the LLV group, with statistically significant differences in all genes except V3 (*p* = 0.077) and p6 (*p* = 0.062; Figure 2D; Supplementary Table S2).

### 3.4 Prevalence and distribution of DRMs

As shown in Table 1, there were no significant differences in the initial ART regimens among virological response groups (*p* = 0.898), with the NRTI+NNRTI regimen predominating across all groups. Compared with the VS group, the VF and LLV groups showed a trend toward increased use of the NRTI+PI/r regimen in their current ART regimens, although the difference did not reach statistical significance. Among the 54 PLWH, 14 exhibited at least one antiretroviral DRM, with the proportions as follows: VF (39.13%, 9/23), LLV (18.18%, 4/22), and VS (11.11%, 1/9). Of these, nine cases exhibited low-level or higher resistance: VF (30.43%, 7/23) and LLV (9.09%, 2/22), while no resistance was detected in the VS group (Table 2).

**Table 2:** Patient characteristics and drug resistance mutations found in proviral DNA.

Group	Sample ID	Viral load (copies/mL)	Previous Therapy	Reason of change	Current Therapy	Drug-resistance mutations in plasma	Drug class	Drug-resistance mutations in provirus	Proportion (Drug resistance level≥2)	Drug resistance level^a^							Association with ART current/ previous^b^
VF	111,102	284,000	EFV + 3TC + TDF	Failure to EFV + 3TC + TDF	AZT/3TC + LPV/r	**T215TA**	NRTI	NO									NO/NO
											DOR	EFV	ETR	NVP	RPV		
							NNRTI	V106I			0	0	0	0	0		
								V179T			0	0	0	0	0		
											BIC	**CAB**	DTG	**EVG**	RAL		
							INSTI	**S147G**	1/6		1	**2**	1	**4**	1		
	111,273	6,690	NO	**/**	EFV + 3TC + TDF					**ABC**	AZT	**FTC**	**3TC**	TDF	**D4T**	**DDI**	YES/—
						**K70E**	NRTI	**K70Q/E**	8/12	**2**	0	1	1	**2**	**2**	**2**	
						**M184V**		**M184V**	8/12	**2**	0	**4**	**4**	0	0	1	
								K70Q/E + M184V		0	0	0	0	1	1	0	
											**DOR**	**EFV**	ETR	**NVP**	**RPV**		
						V179D	NNRTI	V179D			0	1	1	1	1		
						**G190S**		**G190S**	12/12		**2**	**4**	1	**4**	**2**		
								**K103R + V179D**	12/12		0	**2**	0	**2**	**2**		
	111,308	24,100	NO	/	EFV + 3TC + TDF						DOR	EFV	ETR	NVP	RPV		YES/—
						V179E	NNRTI	V179E			0	1	1	1	1		
	111,460	22,000	NO	/	EFV + 3TC + TDF					ABC	**AZT**	FTC	3TC	TDF	**D4T**	DDI	YES/—
						**L210LW**	NRTI	**L210W**	3/4	0	**2**	0	0	0	**2**	1	
											DOR	EFV	ETR	NVP	RPV		
						V179E	NNRTI	V179E			0	1	1	1	1		
								K103R			0	0	0	0	0		
	113,836	323	NO	**/**	AZT/3TC + EFV	K43T					ATV/r	DRV/r	LPV/r	TPV/r	NFV		NO/—
							PI	K43T			0	0	0	1	1		
						**K101E**	NRTI	NO									
						**K103N**											
	114,076	153,000	EFV + AZT + 3TC	Adverse Drug Reaction	AZT/3TC + DTG												YES/YES
			RAL + 3TC + TDF	Economic reason						**ABC**	AZT	**FTC**	**3TC**	TDF	D4T	DDI	
						**M184V**	NRTI	**M184V**	1/1	**2**	0	**4**	**4**	0	0	1	
			EFV + 3TC + TDF	Failure to EFV + 3TC + TDF							DOR	EFV	ETR	NVP	RPV		
			AZT/3TC + LPV/r			V179E	NNRTI	V179E			0	1	1	1	1		
	114,442	233,000	AZT/3TC + EFV	Adverse Drug Reaction	EFV + 3TC + ABC	NO											YES/YES
											DOR	**EFV**	ETR	**NVP**	RPV		
							NNRTI	**K103N**	1/9		0	**4**	0	**4**	0		
			EFV + 3TC + TDF	Adverse Drug Reaction				V106I			0	0	0	0	0		
	116,824	69,000	3TC + TDF	Medical necessity	3TC + DTG + TDF					**ABC**	AZT	**FTC**	**3TC**	TDF	D4T	DDI	YES/YES
						**M184V**	NRTI	**M184V**	3/3	**2**	0	**4**	**4**	0	0	1	
			EFV + 3TC + TDF	Unknown							**DOR**	**EFV**	ETR	**NVP**	**RPV**		
						V106I	NNRTI	V106I			0	0	0	0	0		
						**Y188L**		**Y188L**	3/3		**4**	**4**	1	**4**	**4**		
	118,595	838,000	DTG + 3TC + TDF	Economic reason	3TC/DTG	NO											NO/YES
										ABC	AZT	FTC	3TC	TDF	D4T	DDI	
							NRTI	S68G		0	0	0	0	0	0	0	
			EFV + 3TC + TDF	Adverse Drug Reaction							DOR	**EFV**	ETR	**NVP**	**RPV**		
							NNRTI	**K101E**	1/12		1	**2**	1	**3**	**3**		
									
VS	121,422	<20	NO	/	EFV + 3TC + TDF	/				ABC	AZT	FTC	3TC	TDF	D4T	DDI	NO/—
							NRTI	S68G		0	0	0	0	0	0	0	
LLV	112,389	63	NO	/	3TC/DTG	/					DOR	EFV	ETR	NVP	RPV		NO/—
							NNRTI	V179D			0	1	1	1	1		
	116,167	83	NO	/	EFV + 3TC + TDF	/				ABC	AZT	FTC	3TC	TDF	D4T	**DDI**	YES/—
							NRTI	S68G		0	0	0	0	0	0	0	
								**T69D**	1/1	0	0	0	0	0	1	**3**	
											DOR	EFV	ETR	NVP	**RPV**		
							NNRTI	**E138G**	1/1		0	1	1	1	**2**		
	116,866	29	EFV + 3TC + TDF	Adverse Drug Reaction	BIC/FTC/TAF	/	NNRTI	**V106M**	1/1								NO/YES
						
											**DOR**	**EFV**	ETR	**NVP**	RPV		
											**3**	**4**	0	**4**	0		
			3TC + TDF + LPV/r	Adverse Drug Reaction													
	118,296	74	3TC/DTG + ABT	Unknown	3TC/DTG	/					DOR	EFV	ETR	NVP	RPV		NO/NO
							NNRTI	V179D			0	1	1	1	1		

ABC, abacavir; AZT, zidovudine; FTC, emtricitabine; 3TC, lamivudine; TDF, tenofovir; D4T, Stavudine; DDI, Didanosine; DOR, doravirine; EFV, efavirenz; ETR, etravirine; NVP, nevirapine; RPV, rilpivirine; ATV/r, Atazanavir/r; DRV/r, darunavir/r; LPV/r, lopinavir/r; TPV/r, Tipranavir/r; NFV, Nelfinavir; BIC, bictegravir; CAB, cabotegravir; DTG, dolutegravir; EVG, elvitegravir; RAL, raltegravir.

a Ranking based on resistance levels predicted by the Stanford HIV Drug Resistance Database: 0 = Susceptible; 1 = Potential low-level resistance; 2 = Low-level resistance; 3 = Intermediate-level resistance; 4 = High-level resistance.

b Association with previous therapy shown as “—” if patient did not have previous therapy. The bolded values are indicate that the drug resistance mutation leads to low-level or higher resistance to at least one antiretroviral.

**Figure 3:**
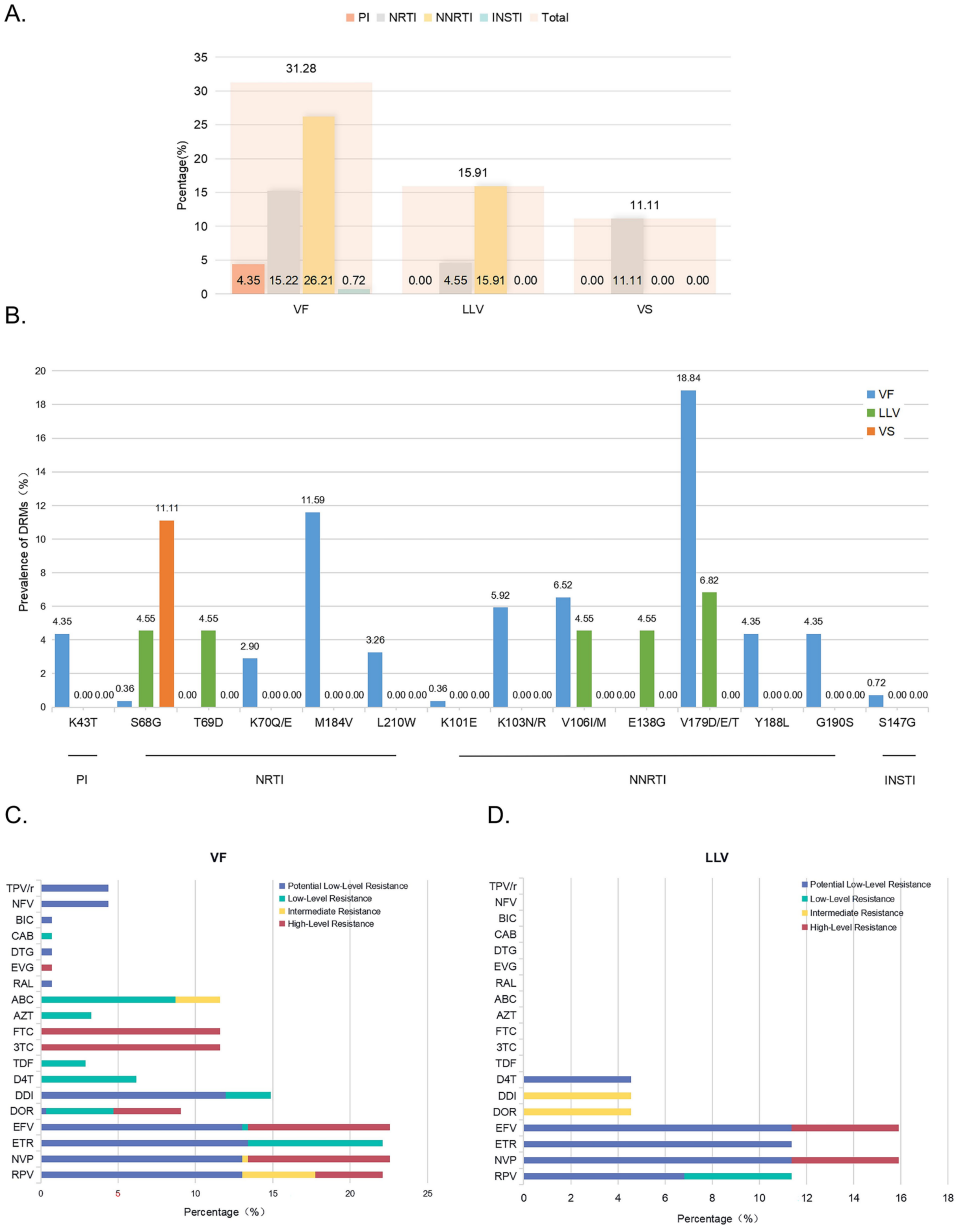
Prevalence of DRMs and resistance levels to various ARVs corresponding to DRMs. **(A)** Prevalence of DRMs in near-full-length quasispecies across groups. **(B)** Prevalence of DRMs for different positions. **(C)** Percentage of resistance levels to various ARVs in VF patients. **(D)** Percentage of resistance levels to various ARVs in LLV patients. DRM, drug resistance mutation; ARVs, antiretrovirals. TPV/r, Tipranavir/r; NFV, Nelfinavir; BIC, bictegravir; CAB, cabotegravir; DTG, dolutegravir; EVG, elvitegravir; RAL, raltegravir; ABC, abacavir; AZT, zidovudine; FTC, emtricitabine; 3TC, lamivudine; TDF, tenofovir; D4T, Stavudine; DDI, Didanosine; DOR, doravirine; EFV, efavirenz; ETR, etravirine; NVP, nevirapine; RPV, rilpivirine.

In the VF group, DRMs were detected in 31.28% of quasispecies, with NNRTIs (26.21%) being most common, followed by NRTIs (15.22%), PIs (4.35%), and INSTIs (0.72%; Figure 3A). The most frequent NNRTI mutations were V179D/E/T (18.84%), V106I/M (6.52%), and K103R/N (5.92%). For NRTIs, M184V (11.59%) was the most common, followed by L210W (3.26%) and K70Q/E (2.90%; Figure 3B). In the LLV group, 15.91% of quasispecies carried DRMs, primarily NNRTIs (15.91%) and NRTIs (4.55%; Figure 3A). The most frequent NNRTI mutations were V179D/E/T (6.82%), V106I/M (4.55%), and E138G (4.55%), while the main NRTI mutations were S68G (4.55%) and T69D (4.55%; Figure 3B). In the VS group, a single DRM (NRTI mutation S68G, 11.11%) was detected.

Further analysis of each drug is summarized in Figures 3C,D. In the VF group, high-level resistance was predominantly observed with 3TC and FTC, followed by NVP and EFV. Intermediate-level resistance was most common with RPV, while low-level resistance was primarily associated with ABC and ETR. Potential low-level resistance was frequently observed with ETR, followed by RPV, NVP, and EFV. In the LLV group, high-level resistance was mainly noted with NVP and EFV, intermediate-level resistance with DOR and DDI, low-level resistance with RPV, and potential low-level resistance with ETR, NVP, EFV, and RPV, in that order. The VS group showed no resistance at any level. Comparison of plasma genotypic resistance testing (GRT) results was performed for the VF group, as viral loads in the other two groups were below 200 copies/mL, precluding plasma GRT (Table 2). Among the 9 PLWH with virological failure, DRMs detected in proviral DNA and plasma RNA samples were generally consistent. However, additional DRMs were identified in 55.56% (5/9) of proviral DNA samples but only in 22.22% (2/9) of plasma RNA samples.

### 3.5 HIV-1 tropism prediction

**Figure 4:**
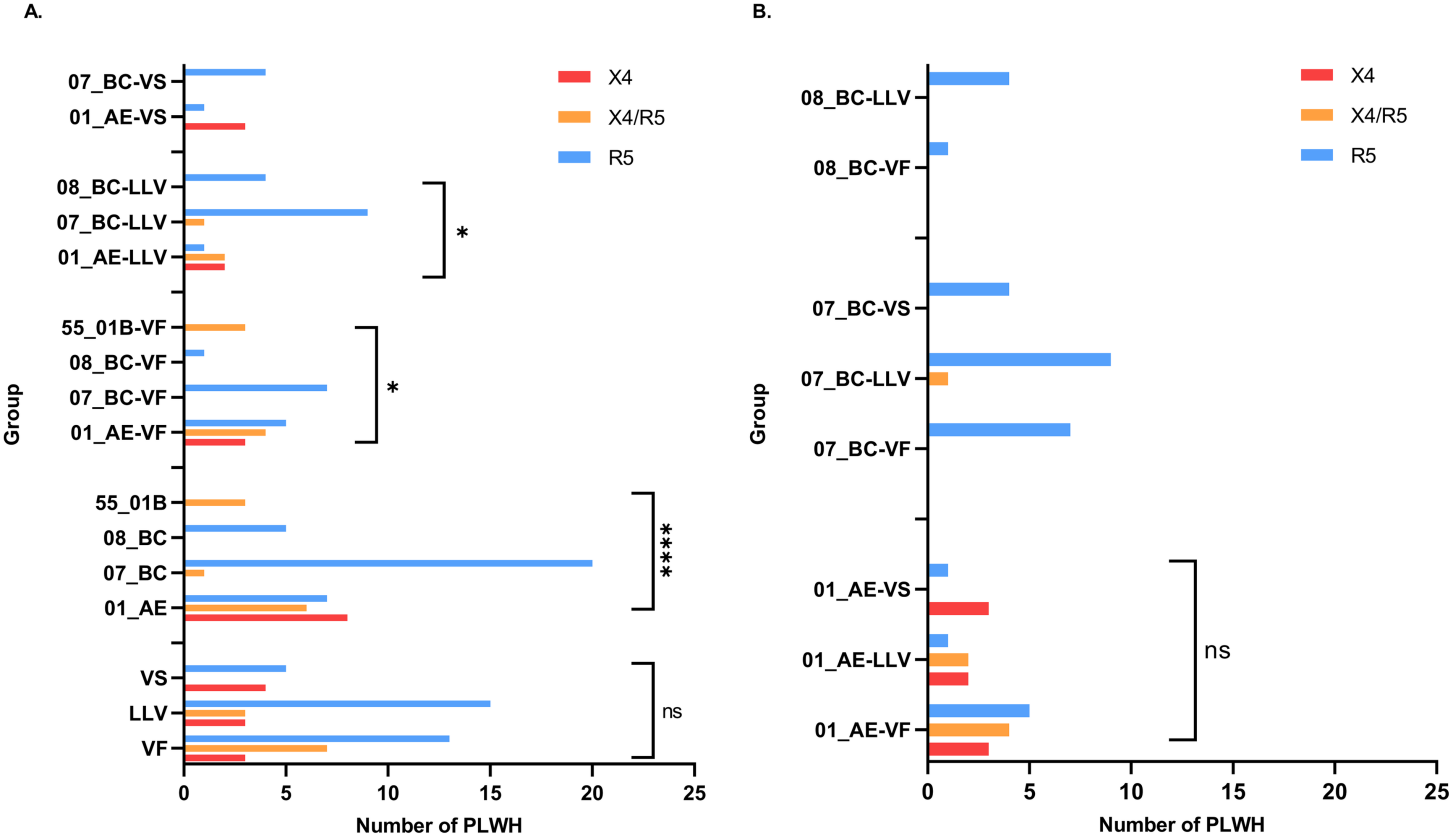
Comparison of coreceptor usage prediction results among different groups. **(A)** Comparison of coreceptor usage prediction results across different virological response groups, various genetic subtypes, and within the same virological response group across different genetic subtypes. **(B)** Comparison of coreceptor usage prediction results among different virological response groups within the same genetic subtype.

X4-tropism analysis of near-full-length quasispecies revealed distinct proportions across the virological response groups: VF (X4 13.0%, X4/R5 30.4%, R5 56.5%; *n* = 23), LLV (X4 14.3%, X4/R5 14.3%, R5 71.4%; *n* = 21), and VS (X4 44.4%, X4/R5 0%, R5 55.6%; *n* = 9). Some PLWH in the VF and LLV groups exhibited proviruses with varying tropisms (Supplementary Table 3). While no significant differences were observed in the pairwise comparisons of tropism population proportions across the three groups, these differences correlated with genetic subtype (Figures 4A,B). Notably, the proportion of X4 and X4/R5 dual-tropism populations was significantly higher in CRF01_AE than in CRF07_BC (*p* < 0.05). Additionally, X4/R5 dual-tropism was significantly elevated in CRF55_01B compared to CRF07_BC (*p* = 0.002) and CRF08_BC (*p* = 0.018).

### 3.6 Amino acids of HIV-1 Gp120 V3 loop in different groups

Comparative analysis of amino acid distributions between groups showed significant differences. In the VF group, Val19 (45.15%), Ala19 (6.21%), Ala22 (30.43%) and Arg22 (54.30%) were predominant. In contrast, the LLV group displayed significantly higher proportions of Ala19 (32.54%) and Ala22 (69.84%), but significantly lower proportions of Val19 (9.52%) and Arg22 (18.65%; *p* < 0.05; Supplementary Table 4). Additionally, significant differences in overall amino acid distribution were observed: between the VF and LLV groups at position 2 (*p* < 0.05), and between the LLV and VS groups at position 27 (*p* < 0.05). At position 5, the VF and LLV groups exhibited a trend toward differential distribution, though it did not reach statistical significance (*p* = 0.054).

## 4 Discussion

The study focused on characterizing near-full-length proviral quasispecies in monocytes from individuals across different virological response groups. In some PLWH within these groups, monocytes harbored intact proviruses, potentially contributing to the establishment, reseeding, and maintenance of viral reservoirs (Fischer-Smith et al., 2001; Crowe et al., 2003; Burdo et al., 2010). Wong et al. (2019) proposed that myeloid progenitor cells in highly vascularized bone marrow may become infected, subsequently differentiating into monocytes that circulate in blood and lymph before infiltrating tissues and differentiating into HIV-infected macrophages, thus establishing tissue reservoirs. HIV-infected monocytes exhibit elevated expression of ALCAM, a connectivity protein that facilitates their entry across the BBB, contributing to HIV-associated neurocognitive disorders (Veenstra et al., 2017). Intermediate monocytes can migrate into tissues, differentiate into non-classical monocytes, and re-enter circulation (Tak et al., 2017), further establishing infection and enabling viral dissemination between tissues. These findings underscore the critical role of monocytes in maintaining replication-competent HIV-1 (Crowe and Sonza, 2000; Veenhuis et al., 2021; Cui et al., 2022), potentially driving virological rebound and viremia. Targeted therapeutic interventions aimed at this reservoir are urgently needed. Notably, the amplification success rates of proviral quasispecies in monocytes followed the order: VF > LLV > VS, indirectly reflecting the relative size of viral reservoirs among the response groups.

Distinct proviral characteristics were observed in the LLV group compared to the VF group, with a higher prevalence of hypermutated sequences. This suggests that host restriction factors, such as APOBEC3F/G-mediated hypermutation, may more effectively limit viral replication and fitness in the LLV population, despite the persistence of low-level viremia. Additionally, we compared the genetic distances between the VF and LLV groups. Although the samples were derived from distinct infection events, the genetic distances between the two groups were comparable, as there were no significant differences in ART duration, time from antibody confirmation to sampling, or transmission routes. Interestingly, the LLV group exhibited greater genetic distances compared to the VF group, suggesting unique evolutionary dynamics under low-level viral replication. The genetic diversity within the LLV group indicates ongoing replication and adaptation, likely driven by selective pressures from ART and immune responses. Previous research has shown that APOBEC3G-induced mutations, while suppressing viral replication, also promote viral sequence diversification, drug resistance mutations, and immune escape (Kim et al., 2010; Monajemi et al., 2014; Campagna et al., 2024), enhancing the virus’s adaptability to host and drug pressures. These findings emphasize the importance of further investigating HIV-1 evolutionary trajectories under varying virological response conditions, particularly in LLV individuals at risk of virological failure.

The detection of DRMs in monocyte-associated proviruses, including intact proviruses, underscores their potential clinical relevance as reservoirs for drug-resistant HIV-1. In resistance analyses, the M184V and V179D/E/T mutations were the most frequently identified in NRTIs and NNRTIs, respectively, within the VF group. In contrast, the LLV group primarily exhibited the S68G and T69D mutations in NRTIs, with M184V notably absent. For NNRTIs, V179D/E/T mutations were also prevalent in the LLV group (Figure 3B). Notably, in the VF group, DRMs were detected in proviral DNA and plasma RNA from the same individual, with no overlap in some cases (e.g., 111,102; Table 2). These differences between proviral DNA and plasma RNA highlight the genetic complexity of the HIV-1 reservoir. Monocytes’ ability to migrate into tissues and differentiate into macrophages further complicates matters, as DRMs in proviruses can persist in tissue reservoirs as long-term archives. Suboptimal antiretroviral penetration in tissue sanctuaries (Cory et al., 2013) facilitates the survival of these mutants, which can re-emerge under insufficient drug pressure or during treatment interruptions. This underscores the value of proviral DNA resistance testing (Chu et al., 2022; Curanovic et al., 2023), which complements routine GRT of plasma RNA or serves as an alternative when viral loads are low, providing a more comprehensive resistance profile to inform clinical decision-making.

HIV-1 X4-tropic viruses are linked to faster disease progression and poorer prognosis. These viruses are associated with accelerated CD4^+^ T cells depletion and heightened immune activation, leading to rapid disease progression in PLWH (Daar et al., 2007; Connell et al., 2020). Notably, X4-tropism was more prevalent in the CRF01_AE subtype than in CRF07_BC (Figure 4A), consistent with previous finding (Hu et al., 2022) demonstrating subtype-associated differences in coreceptor usage and their effects on disease outcomes. The analysis of the gp120 V3 loop amino acid composition across the three study groups revealed significant differences at specific positions. For example, the A19V mutation has been linked to CXCR4 tropism, while the T22A mutation correlates with CCR5 tropism (Dimonte et al., 2011; Abisi et al., 2022). Variations in amino acid distribution at these positions between the VF and LLV groups suggest a higher proportion of X4-tropic viruses in the VF group, aligning with virological response status and the impact of CXCR4-tropic viruses on disease progression. Further research is needed to explore whether variations at other amino acid positions influence functional outcomes.

This study has several limitations. First, technical challenges in performing NFL single-genome amplification (SGA) for HIV-1 DNA, combined with the inherent resistance of monocytes to HIV infection, limited both the number of sequences and participants, particularly in the VS group. Second, the analysis of proviral quasispecies was restricted to sequence-level data, leaving the functionality of intact proviruses unclear, which depends on host factors, immune/drug pressures, and viral characteristics. Third, we lacked comparative data on other HIV-1 reservoir characteristics, such as CD4^+^ T cells and plasma RNA, which are a priority for future research. Fourth, as a cross-sectional study, it cannot comprehensively characterize the dynamics of viral evolution, necessitating the inclusion of longitudinal samples in future research.

In conclusion, monocytes constitute a potentially significant HIV-1 reservoir with distinct genetic and molecular virological features that vary across different virological responses. Near-full-length proviruses were successfully amplified from monocytes in all three virological response groups, with a certain proportion being intact proviruses. The hypermutation rate and genetic distance of HIV-1 proviruses in monocytes from the LLV group are significantly greater than those in the VF group. However, whether this difference directly drives distinct virological responses and the specific extent of its contribution remain unclear, and further research is required for confirmation. Current treatment strategies have largely overlooked the role of non-T-cell reservoirs in HIV-1 persistence. Addressing this gap will require the development of innovative therapeutic approaches to target persistent HIV-1 in these reservoirs, advancing efforts toward a cure.

## Data Availability

The datasets presented in this study can be found in online repositories. The names of the repository/repositories and accession number(s) can be found at: https://www.ncbi.nlm.nih.gov/genbank/, PV207329-PV207526.

## Footnotes

^1^ https://www.hiv.lanl.gov

^2^ https://www.hiv.lanl.gov/content/sequence/BASIC_BLAST/basic_blast.html

^3^ http://www.hiv.lanl.gov/components/sequence/hiv/search/search.html

^4^ https://www.hiv.lanl.gov/content/sequence/GENE_CUTTER/cutter.html

^5^ https://hivdb.stanford.edu

^6^ https://coreceptor.geno2pheno.org/index.php

^7^ https://indra.mullins.microbiol.washington.edu/webpssm/

^8^ https://webs.iiitd.edu.in/raghava/hivcopred/submit.html
